# Whole brain radiation therapy in management of brain metastasis: results and prognostic factors

**DOI:** 10.1186/1748-717X-1-20

**Published:** 2006-06-29

**Authors:** Elisa Y Saito, Gustavo A Viani, Robson Ferrigno, Ricardo A Nakamura, Paulo E Novaes, Cassio A Pellizzon, Ricardo C Fogaroli, Maria A Conte, Joao V Salvajoli

**Affiliations:** 1 Department of Radiation Oncology Hospital do Cancer, Sao Paulo, Brazil

## Abstract

**Purpose:**

To evaluate the prognostic factors associated with overall survival in patients with brain metastasis treated with whole brain radiotherapy (WBRT) and estimate the potential improvement in survival for patients with brain metastases, stratified by the Radiation Therapy Oncology Group (RTOG) recursive partitioning analysis (RPA) class.

**Patients and methods:**

From January 1996 to December 2000, 270 medical records of patients with diagnosis of brain metastasis, who received WBRT in the Hospital do Cancer Sao Paulo A.C. Camargo in the period, were analyzed. The surgery followed by WBRT was used in 15% of patients and 85 % of others patients were submitted at WBRT alone; in this cohort 134 patients (50%) received the fractionation schedule of 30 Gy in 10 fractions. The most common primary tumor type was breast (33%) followed by lung (29%), and solitary brain metastasis was present in 38.1% of patients. The prognostic factors evaluated for overall survival were: gender, age, Karnofsky Performance Status (KPS), number of lesions, localization of lesions, primary tumor site, surgery, chemotherapy, absence extracranial disease, RPA class and radiation doses and fractionation.

**Results:**

The OS in 1, 2 and 3 years was 25, 1%, 10, 4% e 4, 3% respectively, and the median survival time was 4.6 months. The median survival time in months according to RPA class after WBRT was: 6.2 class I, 4.2 class II and 3.0 class III (p < 0.0001). In univariate analysis, the significant prognostic factors associated with better survival were: KPS higher than 70 (p < 0.0001), neurosurgery (p < 0.0001) and solitary brain metastasis (p = 0.009). In multivariate analysis, KPS higher than 70 (p < 0.001) and neurosurgery (p = 0.001) maintained positively associated with the survival.

**Conclusion:**

In this series, the patients with higher perform status, RPA class I, and treated with surgery followed by whole brain radiotherapy had better survival.

This data suggest that patients with cancer and a single metastasis to the brain may be treated effectively with surgical resection plus radiotherapy. The different radiotherapy doses and fractionation schedules did not altered survival.

## Background

Brain metastases represent an important cause of morbidity and mortality, and are the most common intracranial tumors in adults, occurring in approximately 10% to 30% of adult cancer patients [1]. The risk of developing brain metastases varies according to primary tumor type, with lung cancer accounting for approximately one half of all brain metastases [2]. The prognosis of patients with brain metastases is poor; the median survival time of untreated patients is approximately 1 month [3]. With treatment, the overall median survival time after diagnosis is approximately 4 months [4]. The Radiation Therapy Oncology Group (RTOG) recursive partitioning analysis (RPA) describes three prognostic classes, defined by age, Karnofsky Performance Score (KPS), and disease status [5]. The most widely used treatment for patients with multiple brain metastases is WBRT. The appropriate use of WBRT can provide rapid attenuation of many neurological symptoms, improve quality of life, and is especially beneficial in patients whose brain metastases are surgically inaccessible or when other medical considerations remove surgery from the list of appropriate options [6,7]. The use of adjuvant WBRT after resection or radiosurgery has been proven to be effective in terms of improving local control of brain metastases, and thus, the likelihood of neurological death is decreased [8].

The majority of patients who achieve local tumor control die from progression of extracranial disease, whereas the cause of death is most often due to CNS disease in patients with recurrent brain metastases [7,8]. There is not currently consensus on the optimal radiation schedule for patients with brain metastases. Standard treatment regimens include all of the dose ranges evaluated in the early RTOG studies, and is dependent upon issues such as the severity of CNS symptoms, the extent of systemic disease, and physician preference. In this cohort, we evaluated the prognostic factors and the importance of RPA classification (RTOG) for survival in patients with diagnosis of brain metastasis, who receive WBRT alone or postoperative.

## Materials and methods

The records of 270 patients with brain metastases, who were treated with WBRT at our institution between January 1996 and December 2000, were analyzed retrospectively.

At diagnosis of brain metastasis, the follow variables were analyzed for survival: age, sex, location of brain metastasis, primary tumor type, and extent of disease, initial Karnofsky score, dose and fractionation radiotherapy schedule, surgery, chemotherapy and RPA class, showed in table 1. The supportive care (oral prednisone) and neurological status was not evaluated. Chemotherapy was administered to the patients with systemic disease in activity after WBRT. Brain metastases were detected by contrast-enhanced cerebral computed tomography (CT) or magnetic resonance imaging (MRI). WBRT was performed in all patients with cobalt 60 gamma rays or with 4 MV photons of a linear accelerator. The whole brain was irradiated by usual bilateral fields that encompassed the cranium with a 1 cm margin. Individual shielding blocks were fabricated for all patients, when necessary. The total dose was 30–40 Gy, with a median of 35 Gy, in daily fractions of 2.0–3.0 Gy. During the study period two fractionation schemes were used: conventional fractionation with daily fractions of 2 Gray (Gy), five days per week to a planned total dose of 40 Gy (n = 102) and hypofractionation with daily fractions of 3 Gy, five days per wk to a planned total dose of 30 Gy (n = 134). The surgical resection was indicated in single brain metastases with diameter less or equal than 3 cm, favorable localization and control systemic disease. The supportive care (prednisone oral) was introduced in begin of treatment or during radiotherapy. The recursive partitioning analysis (RPA) was used to classify the patients with brain metastases. Class I contained all patients with a Karnofsky performance status (KPS ≥ 70, age < 65 years, controlled primary tumor and no extracerebral metastases), Class III contained patients with a KPS <70, and Class II contained all other patients, showed in table 1.

**Table 1:** characteristic of treatment and patients

**AGE**	median	range
Patients	57	38 – 82
		
**SEX**	number	%
MALE	111	41.1
FEMALE	159	58.9
		
**KPS**	number	%
< 70	154	57
>= 70	115	42.6
		
**NEUROSURGERY**	number	%
YES	41	15.2
NO	229	84.8
		
**DOSE(Gy) FRACTIONATION (fr)**	number	%
40 Gy/20 fr	102	37.8
30 Gy/10 fr	134	49.6
OTHERS	34	12.6
		
**NUMBER LESIONS**	number	%
SINGLE	103	38.1
MULTIPLE	161	59.6
		
**CHEMOTHERAPY**	number	%
YES	54	20
NO	214	79.2
		
**RPA CLASS**	number	%
CLASS I	42	15.5
CLASS II	72	26.6
CLASS III	151	55.9
		
**LOCALIZATION**	number	%
SUPRATENTORIAL	140	51.9
INFRATENTORIAL	24	8.9
BOTH	47	17.4
		
**PRIMARY DISEASE CONTROL**		
YES	141	52.2
NO	121	44.8
		
**EXTRA CRANIAL METASTASIS**		
YES	178	65.9
NO	92	34.1

### Statistical analysis

All patients alive at the time of analysis were censored with the date of last follow-up. The endpoint of the study was overall survival. Survival was calculated from the first day of radiotherapy using the method of Kaplan Meier. Survival curves were compared using the log-rank test. The covariates examined in all cases were: age, sex, location of brain metastasis, primary tumor type, extent of disease, initial Karnofsky score, dose and fractionation radiotherapy schedule, neurosurgery and RPA class. All factors with a P-value ≤ 0.05 at univariate analysis were entered into a multivariate analysis using the proportional hazards model (Cox Regression) with confidential interval of 99%.

## Results

**Figure 1:**
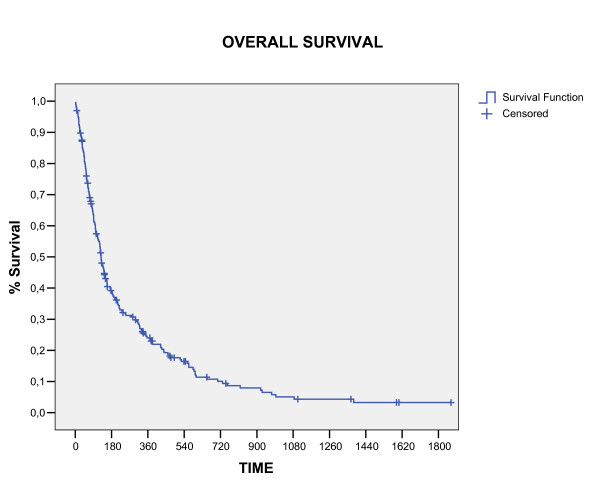
Overall Survival (Kaplan Meier).

**Figure 2:**
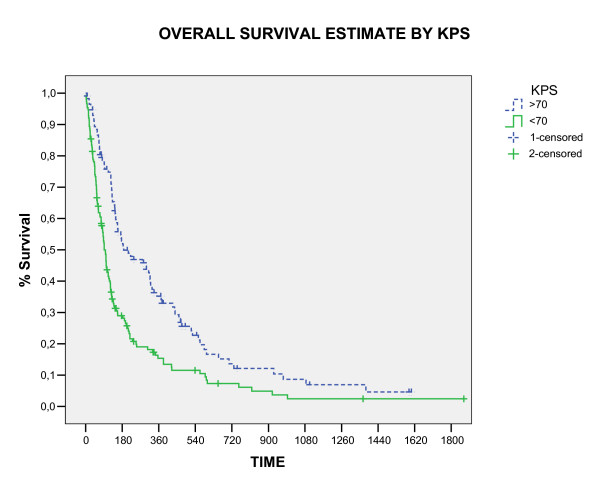
Overall Survival by KPS (Log Rank).

**Figure 3:**
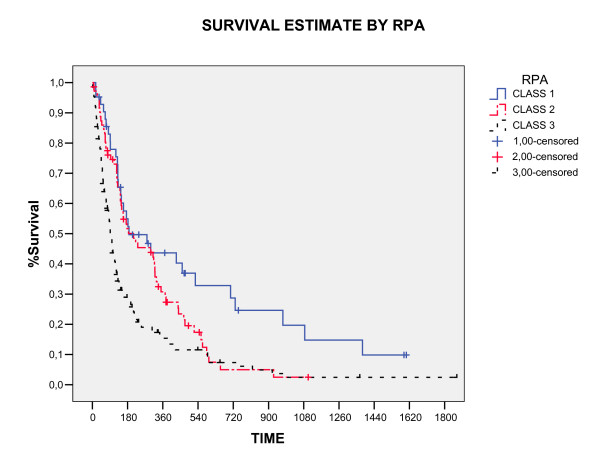
Overall Survival by RPA CLASS (Log Rank).

The overall survival rate in 1, 2 and 3 years was 24%, 9.4%, and 4.3%, respectively (figure 1). Three patients were alive in moment of this analysis with a median survival time of 4.42 years (range, 3.8 – 5.1). All these patients had single brain metastasis, high KPS, cranial extra disease controlled and were submitted to neurosurgery before WBRT. The median survival time for all the studied patients was 4.6 months (CI 95% 3.7 – 6, 4). The RPA class analysis showed strong relation with survival (p < 0.0001) and the median survival time by RPA class in months was: class I 6.2, class II 4.2 and class III 3.0. The significant prognostic factors associated with better survival were: higher KPS (p < 0.0001), neurosurgery (P < 0.0001) and single metastases (p = 0.009), showed in table 2 and figure 2, 3, 4. In multivariate analysis, the factors associated positively with survival were: neurosurgery (p = 0.001, HR = 2, CI99% = 1.2–3.3) and KPS higher than 70 (p < 0.001, HR = 1.56, CI99% = 1.19–2.04), demonstrated in table 3.

**Table 2:** univariate analysis of significant factors for survival (Log Rank Test)

**Variable**	**number**	**%**	**% OS 12 MONTHS**	**P**
**AGE**				
< 65 YEARS	195	72.3	22.6	0.84
>= 65 YEARS	75	27.7	28	
**SEX**				
MALE	111	41.1	23	0.17
FEMALE	159	58.9	25.5	
**KPS**				
< 70	154	57	15.4	<0.0001
>= 70	115	42.6	35.2	
**NEUROSURGERY**				
YES	41	15.2	49.2	<0.0001
NO	229	84.8	19.2	
**DOSE(Gy) FRACTIONATION (fr)**				
40 Gy/20 fr	102	37.8	27.3	0.12
30 Gy/10 fr	134	49.6	24.7	
OTHERS	34	12.6		
**NUMBER LESIONS**				
SINGLE	103	38.1	33.4	0.009
MULTIPLE	161	59.6	17.9	
**CHEMOTHERAPY**				
YES	54	20	36.8	0.09
NO	214	79.2	20.7	
**RPA CLASS**				
CLASS I	42	15.5	43.6	<0.0001
CLASS II	72	26.6	30.8	
CLASS III	151	55.9	15.3	
**LOCALIZATION**				
SUPRATENTORIAL	140	51.9	27	0.29
INFRATENTORIAL	24	8.9	18	
BOTH	47	17.4	25.2	
**PRIMARY DISEASE CONTROL**				
YES	141	52.2	30.1	0.06
NO	121	44.8	20.2	
**EXTRA CRANIAL METASTASIS**				
YES	178	65.9	23.3	0.09
NO	92	34.1	28.6	

**Table 3:** Multivariate Analysis of significant factors for survival (Cox Regression)

**VARIABLE**	**P**	HR *	**99% confidential interval**	
*NEUROSURGERY*				
*YES*	*0.001*	*2*	*1.2*	*3.3*
*NO*		1(REF)**		
*SINGLE METASTASES*				
YES	*0.48*	*1.11*	*0.75*	*1.23*
NO		*1(REF)*		
*KPS*				
*> = 70*	*<0.0001*	*1,56*	*1.19*	*2.04*
*< 70*		*1(REF)*		

*HR = hazard risk

**REF = value reference

**Figure 4:**
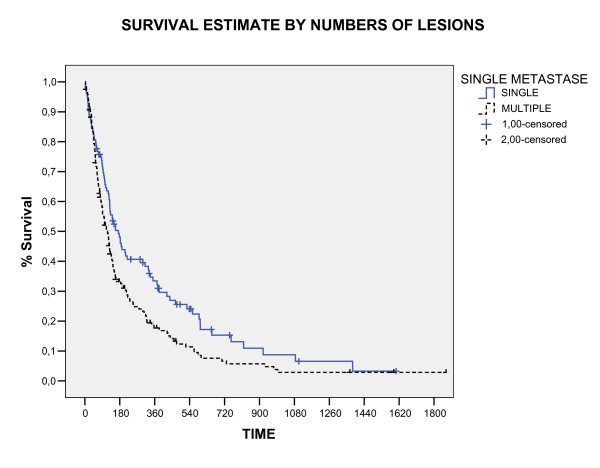
Overall Survival by number of lesions (Log Rank).

## Discussion

Brain metastases are the most common form intra cranial tumor accounting significantly more than one- half of brain tumors in adults. Because of advanced in the diagnoses and management of this condition, most patients receive palliative treatment and majorities don't die from metastases. In this cohort, we evaluate patients with brain metastasis, multiples or solitaries lesions, who receive WBRT alone or WBRT after surgical resection of lesion.

The goal of postoperative WBRT in patients with solitary brain metastasis is to destroy microscopic residual cancer cells at the site of resection and others localizations within the brain. Until recently, the value of this approach was derived exclusively from retrospective studies[8,11,12]. Several of this studies found that adjuvant WBRT reduced the recurrence rate and two studies demonstrated prolong survival[12,13]. One randomized trial has examined the role of pos operative WBRT in patients with single metastasis[13]. In this study patients who received radiation were significantly less likely to fail in the brain(18% vs 70%) e were significantly less likely to die of neurological causes. In our series, patients submitted at resection plus WBRT were significantly less likely to die (p = 0,001), mainly the patients with solitary metastasis and higher KPS.

The Radiation Therapy Oncology Group (RTOG) has attempted to determine the optimal dose fractionation schedules for patients with brain metastasis in various randomized trials [9-11]. All these trials have failed to show any benefit in survival for different doses and fractionation schedules of treatment. In this cohort, 40 Gy in 20 fractions or 30 Gy in 10 fractions, were not associated with any benefit to survival. (p = 0,8). The according with our data, patients with good prognosis (RPA class I) who are likely to survive more than six months, such as those with single metastasis with controlled systemic disease, should be treated with prolonged fractionation to decreased the likelihood of late CNS toxicity.

The end point of this cohort was to evaluate the different prognostic factors related with overall survival and to analyze the importance of recursive partitioning analysis (RPA) class (RTOG) in patients with brain metastasis. In our data, the prognostic factors associated with better survival were: Higher KPS (p < 0.0001), solitary metastasis (p = 0.009), resection of lesion (p = 0.0001) and RPA class I (p = 0.0001), all these prognostic factors were showed for others authors. [8,14,15,17,18] The others factors (age, gender, chemotherapy, dose and fractionation schedule) analyzed were not associated with any effect in survival. RPA class in this study showed similar results to RTOG protocols [5], with the median survival time for class I (6.2 months), II (4.2 months) and III (3.0 months) (p = 0.0001), respectively. This data demonstrate that the use of RPA class may identify patients most likely to benefit from treatment and allow new therapies to be evaluated on homogeneous patient groups.

In this study, patients with multiple brain metastases that received WBRT had poorer survival than patients with single brain metastases (P = 0.0001). We did not evaluate the use supportive care (oral predinisone) plus radiotherapy versus supportive care alone or WBRT alone versus supportive care. However, Horton et al. [19] compared WBRT plus supportive care (oral prednisone) versus supportive care alone. Median survival in the prednisone alone arm was 10 weeks compared with 14 weeks in the combined arm (p-value not stated). The proportion of patients with an improvement in performance status was similar in the prednisone- alone and the combined WBRT and prednisone arms (63% versus 61%, respectively). Data on tumor response, intracranial progression-free duration, quality of life, and toxicity were not reported.

In our study no patients received Radiosurgery (SRS); however, a larger recently published trial (RTOG 95-08) [20] provides compelling evidence for the use of SRS boost following WBRT in patients with newly diagnosed one to three brain metastases. In the RTOG 95-08, SRS after WBRT has been validated with level 1 evidence as a standard of care option in the management of patients with single brain metastases.

In other recently published prospective randomized Japanese trial, JROSG 99-1, patients were randomly assigned to SRS alone, versus WBRT and SRS. The actuarial 6 month freedom from new brain metastases was 48% in the SRS alone arm, and 82% in the SRS and WBRT arm (P = 0.003). Actuarial 1 year brain tumor control rate for the lesions treated with SRS was 70% in the SRS alone arm and 86% in the SRS and WBRT arm (P = .019) [21]. Clinical trial-based assessments therefore suggest high rates of intracranial failures and reduced local control rates when WBRT is omitted or delayed.

In conclusion, WBRT continues to be an efficacious treatment in the management of brain metastasis. Patients with RPA class I may be effectively treated with local resection or radiosurgery followed by WBRT, mainly in those patients with single metastases, higher KPS and cranial extra disease controlled. Despite the use of WBRT, outcomes are poor and efforts should be made to incorporate multimodality approaches including surgery, radiosurgery, chemotherapy, and radiotherapy sensitizers to improve survival.

**Figure 5:**
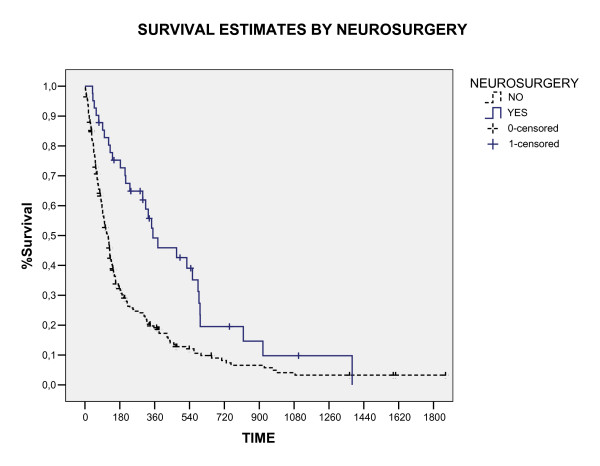
Overall Survival by neurosurgery (Log Rank).
